# Persistent Activity in Neural Networks with Dynamic Synapses

**DOI:** 10.1371/journal.pcbi.0030035

**Published:** 2007-02-23

**Authors:** Omri Barak, Misha Tsodyks

**Affiliations:** 1 Department of Neurobiology, The Weizmann Institute of Science, Rehovot, Israel; University College London, United Kingdom; 2 Group for Neural Theory, Ecole Normale Supérieure and Collège de France, Paris, France

## Abstract

Persistent activity states (attractors), observed in several neocortical areas after the removal of a sensory stimulus, are believed to be the neuronal basis of working memory. One of the possible mechanisms that can underlie persistent activity is recurrent excitation mediated by intracortical synaptic connections. A recent experimental study revealed that connections between pyramidal cells in prefrontal cortex exhibit various degrees of synaptic depression and facilitation. Here we analyze the effect of synaptic dynamics on the emergence and persistence of attractor states in interconnected neural networks. We show that different combinations of synaptic depression and facilitation result in qualitatively different network dynamics with respect to the emergence of the attractor states. This analysis raises the possibility that the framework of attractor neural networks can be extended to represent time-dependent stimuli.

## Author Summary

Imagine driving a car when you hear the sentence, “Take the next left.” Immediately, auditory “left” neurons begin to fire. But to use this information a few seconds later when you reach the junction, these neurons should persist in their firing after the auditory stimulus has been removed. This persistent activity, believed to be the basis for working memory, is maintained by a recurrent neural network in memory-related cortical areas. Previous studies showed that a network can maintain memories of “which” stimulus was observed. It has recently been shown that synapses between excitatory cells in the prefrontal cortex, where persistent activity is often observed, exhibit activity-dependent dynamics. Different forms of synaptic dynamics such as depression and facilitation were observed. In this work, we use a mathematical model of a recurrent neural network to analyze the effect of introducing dynamic synapses in the context of persistent activity. We find that the initiation of persistent firing can depend on the duration of the input. These results open the possibility that recurrent neural networks can encode not only “which” stimulus was observed, but also for “how long.”

## Introduction

Working memory enables us to hold the trace of a fleeting stimulus for a few seconds after it is gone, thus enabling the manipulation of information over time. Recordings from neurons in monkeys performing working memory tasks reveal stimulus-selective spiking activity that persists after the removal of the stimulus (see, e.g., [1–4]). These persistent activity states (attractors) are considered to be the neuronal substrate of working memory [5].

The sustained persistent activity is believed to be achieved by excitatory interpyramidal connections that are either prewired or formed during the learning of the task [3] (see also [6,7] for a possible role of single-cell mechanisms). In vitro studies of such connections in the cortex revealed pronounced short-term plasticity effects [8]. In the sensory areas of the cortex, the dominant effect is synaptic depression, expressed as a rapid decay of synaptic efficacy following the presynaptic firing [9]. Several theoretical studies investigated the effects of synaptic depression on the existence and stability of attractor states (see, e.g., [10,11]). Wang et al. [12] recently performed experiments to investigate short-term synaptic plasticity in the prefrontal cortex, one of the cortical areas where persistent activity is observed [4]. They found that interpyramidal connections in this area exhibit various degrees of synaptic facilitation, with three different classes of connections identified. While synaptic facilitation was recently mentioned as a stabilizing factor for network attractors [13], there is as yet no systematic study of its effect on the dynamics of recurrent neural networks undergoing the transition from background to persistent states after the presentation of a stimulus.

**Figure 1:**
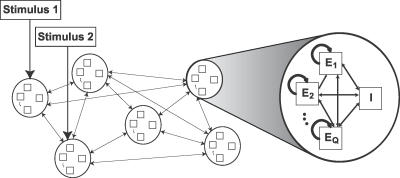
Network Structure The network is divided into several populations, each responding primarily to a certain stimulus. Each population is further partitioned into subpopulations, differing in their synaptic properties. Connections are strongest within subpopulations, weaker between subpopulations, and weakest across populations.

In this contribution, we consider an attractor neural network with connections that have already been formed by learning several stimuli [14,15]. We assume that the network comprises a set of neuronal populations, each responding primarily to a certain stimulus. This scheme, via Hebbian learning, can strengthen the synaptic connections within a population and form a stable activity state. Drawing on recent experimental results [12], we assume that the neurons within each population differ in the dynamic properties of their synapses and thus exhibit different temporal response profiles to the same stimuli. This firing can then lead to a further differentiation of synaptic strengths within the population, whereby neurons with similar synaptic dynamics are connected more strongly to one another than to ones with dissimilar synaptic dynamics. We thus consider a network comprising several attractor populations, each divided into subpopulations with different synaptic dynamics (Figure 1). These populations interact via both excitatory dynamic synapses and inhibition to generate rich dynamics in response to external stimuli. Since the synaptic dynamics differ between subpopulations, we expect them to respond differently to different temporal profiles of the input, which could result in a greater computational power for the network.

## Results

We consider a neural network with a sparse representation of external stimuli. This means that a given stimulus targets a population that is a small fraction of the network, and therefore there is a very small overlap between the populations. As described in the Introduction, we assume that long-term learning processes result in a three-tier connectivity structure in the network. A subpopulation of neurons within a given population that share similar short-term synaptic dynamics develop the strongest connections; neurons within a population but differing in their synaptic parameters have weaker connections, and neurons of different populations have the weakest connections. We simplify the system by assuming homogeneous short-term synaptic dynamics within each subpopulation. This allows us to derive a set of rate equations for subpopulations (see Methods), thus greatly simplifying the analysis.

### Dynamics of a Single Subpopulation with Increasing Facilitation Levels

We begin our analysis with a single homogeneous excitatory subpopulation that is amenable to analytical treatment. The analysis was performed using a firing rate model (see, e.g., [16]) with the mean synaptic current, *h,* and mean firing rate, *R.* We combine the current dynamics with the model of dynamic synapses introduced in [17,18]. The synaptic feedback is characterized by the set of four parameters: *J* (absolute efficacy), *U* (initial utilization parameter, analogous to release probability), and *t_f_* and *t_r_* (time course of facilitation and depression, respectively). Briefly, the running value of the utilization parameter, *u,* is facilitated every time a spike arrives, and decays to its baseline level, *U,* with the time constant *t_f_*. Correspondingly, the running fraction of neurotransmitter available, *x,* is utilized by each spike in proportion to *u* and recovers to its baseline value of 1 with the time constant *t_r_.* The system dynamics are therefore described by the following three differential equations (see Methods):

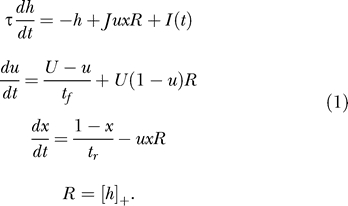



Here, *I*(*t*) is the external input relative to threshold, and τ is the decay time constant of the synaptic current. *R* is the average population firing rate, which is assumed to be a threshold-linear function of synaptic current:

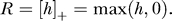
The term *Jux* in the first equation for the synaptic current reflects the effect of synaptic short-term dynamics. The second equation describes a facilitation process that determines the running value of *u,* which in turn enters into the third equation for the depression process.

**Table 1:**
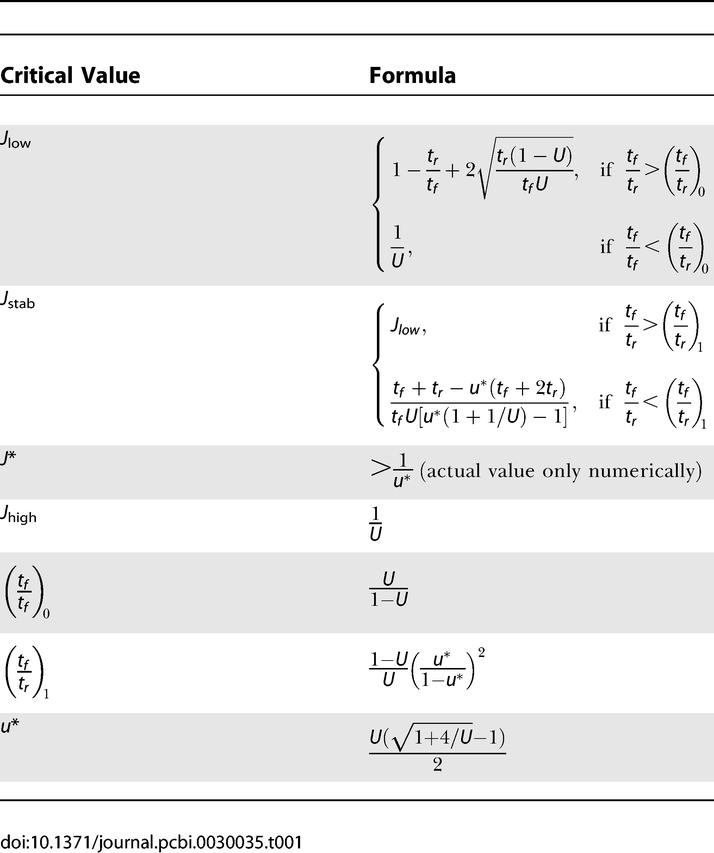
Critical Values of Parameters Separating Regimes of Different Qualitative Behavior (See Text and Figure 6)

**Figure 2:**
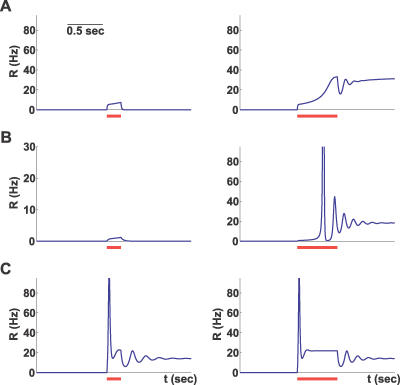
Network Dynamics in Response to Transient Stimuli for Three Different Facilitation Levels The three rows use parameter sets A, B, and C, respectively, with facilitation strongest in A and weakest in C (see Methods, Table 2). The red bars mark two stimulus durations of 200 and 700 ms for short and long bars, respectively. The stimulus magnitude is 4 Hz for A and C, and 0.2 Hz for B.

The combination of the three kinetic parameters, *t_f_*, *t_r_*, and *U,* can describe widely different synaptic behaviors: from strong depression (*t_r_* ≫ *t_f_* and relatively high values of *U*) to strong facilitation (*t_f_* ≫ *t_r_* and small values of *U*). In fact, three different groups of synapses were identified in the prefrontal cortex [12], two corresponding to these extreme cases, and an intermediate one with *t_f_* ≈ *t_r_.* We therefore simulated the network equations for three different sets of synaptic parameters that roughly correspond to these observed synaptic groups (see Methods). For each network, we chose the minimal connection strength, *J,* that enables a persistent state, and subjected the networks to a transient input of two different durations. As can be seen in Figure 2, all three networks could be driven to a persistent state that outlasts the input, but the dynamics of approaching the persistent state and the effect of input duration are qualitatively different between the networks. Most notably, the facilitating network (Figure 2A) requires a certain minimal input duration and approaches the persistent state gradually, while the depressing network (Figure 2C) responds quickly with a large transient increase in the population firing rate. This transient response, called a “population spike,” reflects a near-coincident firing of a large number of neurons (as known from the previous studies [19,20]). The network with the intermediate set of parameters displays a delayed population spike and strong transient oscillations (Figure 2B).

**Table 2:**
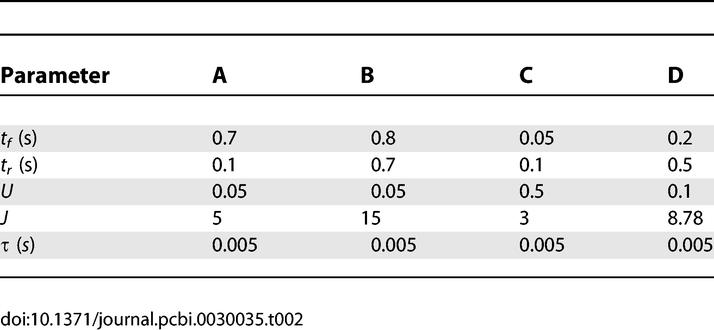
Four Different Parameter Sets (A, B, C, and D) Used in Numerical Simulations throughout the Paper

In the next sections, we present a more thorough analysis of the network dynamics, with an emphasis on the full repertoire of behaviors for different values of synaptic parameters. These three examples can then be seen as particular cases of a general scheme.

### Steady State: Recurrent Excitation Leads to Bistability

**Figure 3:**
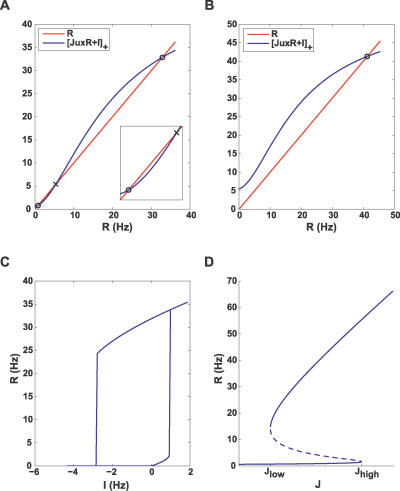
Steady State Analysis (A,B) Steady state values of the firing rate shown as intersection points (circle, stable; cross, unstable) between the decay and recurrent excitation terms in Equation 2, for low, 0.5 Hz (A), and high, 5.5 Hz (B) external current. (C) Hysteresis plot showing stable steady states for different values of input. (D) Bifurcation diagram for *I* = 0.5 Hz, illustrating the steady states for different values of connection strength. The dashed line marks unstable equilibria. All subplots use parameter set A (see Methods, Table 2).

**Figure 4:**
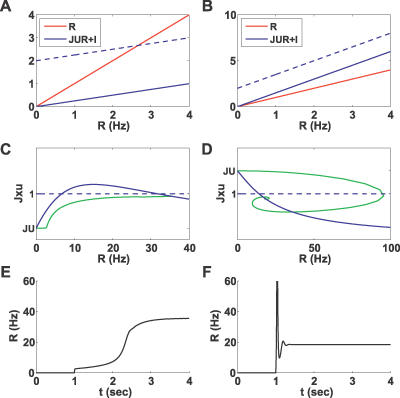
Fast Dynamics Left and right columns use parameter sets A and C, respectively (see Methods, Table 2). (A,B) Steady state analysis similar to Figure 3, but with *x*,*u* frozen at their resting values (see Equation 4). The dashed line illustrates recurrent excitation after an external input is increased. Note that only in (A) does a steady state remain. (C,D) Steady state value of *Jux* as a function of *R* (solid blue line) overlaid with the condition for persistent activity *Jux* = 1 (dashed blue line) and the trajectory caused by current increase (green line). (E,F) Time course of the firing rate for both cases.

The firing rate of a network without recurrent excitation will decay once external input is removed. Recurrent excitation provides a positive feedback that, if powerful enough, can balance this decay even in the lack of external input and sustain persistent activity. The balancing condition is given by the steady state equation for the population firing rate obtained from Equation 1:

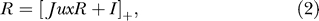
where the steady state value of *ux* depends on *R* as stationary solutions of the second and third equations of Equation 1 (see Equation 15 in Methods). Figure 3 illustrates graphically the solutions of the steady state equation. Figure 3A and 3B shows the balance between the decay term and the recurrent excitation term for two input levels. For a small input, the system has three steady state solutions, the lowest representing a spontaneous low-activity state, and the highest representing a persistent state (Figure 3A). The intermediate solution is always unstable. The presence of the low-activity state is due to the facilitation that results in the initial increase in the effective connection strength *Jux* with *R,* until depression takes over for higher *R* (Figure 4C and 4D, solid blue lines; see also [17]). This initial facilitation leads to the corresponding increase in the slope of the effective excitation as the network activity increases (inset in Figure 3A). The minimal facilitation time constant *t_f_* that is needed for this regime to be observed can be computed (see Methods, after Equation 16):

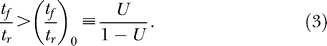
For smaller values of *t_f_*/*t_r_*, the facilitation effect is not observed, and the effective connection strength monotonically decreases with the activity rate *R* (Figure 4D).

Increasing the input beyond a certain level leaves the network with the persistent state only (Figure 3B). This analysis is summarized in Figure 3C, showing the steady states of the network for different values of input. It follows that the persistent state can be reached by temporarily increasing the input to the level where the spontaneous state disappears and then reducing it back to the bistable regime (hysteresis).

**Figure 5:**
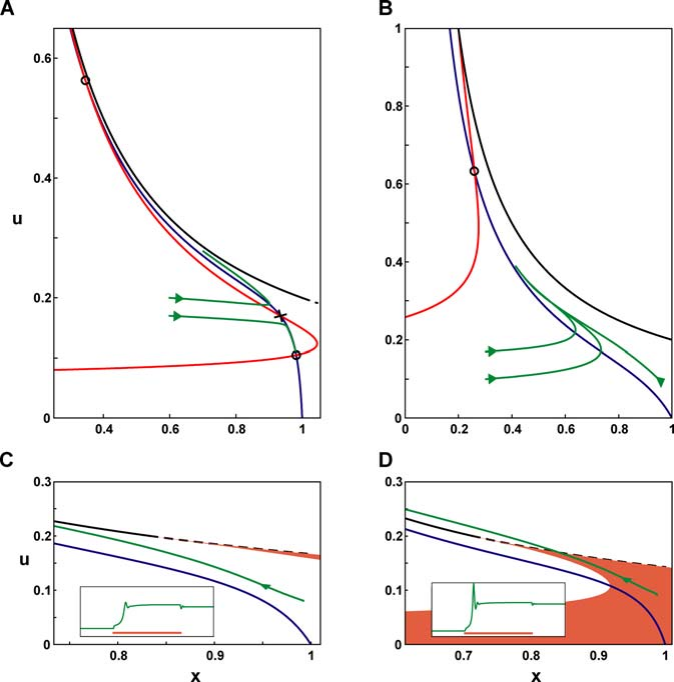
Slow Dynamics on the *x*–*u* Phase Plane The *x* nullcline, the *u* nullcline, and the forbidden line (*Jux* = 1) are depicted in blue, red, and black, respectively. Simulated trajectories (performed in 3-D and projected onto 2-D) are in green. The attractive part of the forbidden line is shown as a dashed line, and the repulsive part as a solid line. (A) For a small input (*I* = 0.85 Hz), the network has three steady states; circles indicate the stable steady states, and crosses indicate the unstable ones. (B) For a high input (*I* = 8 Hz), the network has only one steady state. (C,D) Shaded area is the forbidden line's basin of attraction. Insets show *R*(*t*) for displayed trajectories. *J* is below and above J* for (C) and (D), respectively, leading to a smooth transition in (C) and a population spike in (D). In all plots, parameter set A is used (see Methods, Table 2), except for *J* = 6 in (C) and *J* = 7 in (D).

Steady states of the network for a small positive input and different values of *J* are shown on Figure 3D. As mentioned above, for the persistent state to exist, the recurrent excitation has to be powerful enough, *J* > *J*_low_. The value of *J*_low_ can be calculated from the first and last equations of Equation 1 by observing that *R* can only be nonzero while *I* = 0 when *Jux* = 1 at the steady state (dashed lines in Figure 4C and 4D). When facilitation is strong (inequality (3) holds), this requirement can be met if *J*(*ux*)*_p_* > 1, where (*ux*)*_p_* is the peak steady state value of *ux* as a function of *R.* This means that *J*_low_ = 1/(*ux*)*_p_* and can be computed from the model equations (see Table 1 and Methods, Equation 16). When *J* is increased beyond *J*_high_ = 1/*U,* the low-activity steady state disappears, and the system is no longer bistable. Note that for a depressing system, where *ux* is a monotonically decreasing function of *R, J*_low_ = *J*_high_ = 1/*U.* In this case, the system can only be bistable for *I* < 0, with one of the steady states having zero firing rate.

**Figure 6:**
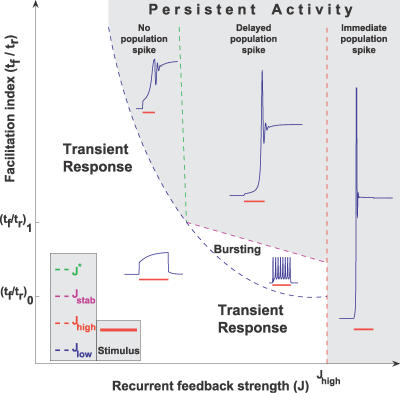
Summary of Analysis for a Single Subpopulation Five regions in parameter space with qualitatively different network behavior are illustrated. The traces shown in blue were obtained with the following parameter sets (clockwise, beginning from Transient Response): A with *J* = 0.8*J*_low_, A, A with *J* = 1.1J*, A with *J* = 1.1*J*_high_, D (see Methods, Table 2).

Finally, the persistent state that appears at *J* = *J*_low_ is not necessarily stable. The analytical condition for stability is hard to obtain in the full system, and we therefore address it below in the framework of a reduced 2-D system.

### Dynamics of Transition to the Persistent State

To analyze the temporal dynamics of the network after the change in the input, we use the fact that the time constant of the current dynamics in Equation 1, *τ,* is on the order of a few milliseconds. This is usually much smaller than the depression and facilitation time constants, thus enabling a separation of timescales between the slow variables *x,u* and the fast variable *h.* This means that *x* and *u* can be regarded as being constant when considering the fast dynamics of *h* and *R* on the timescale of *τ.* Conversely, *R* and *h* can be approximated to be at the steady state (if one exists) when the slower *x*,*u* dynamics are considered.

#### Fast dynamics.

To explain the differences in the immediate response of networks with strong facilitation and depression (Figure 2), we first consider the initial fast dynamics of *R* after the sudden increase in the input. The slow variables can be considered to remain at their rest values (*x* ≅ 1, *u* ≅ *U*). Once *x*,*u* are fixed at these values, the dynamics of *R* are governed by the simple equation:

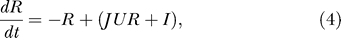
where we set *R* = *h* since we are dealing here with positive inputs.

Figure 4A and 4B depicts the balance between the decay (red line) and the recurrent excitation terms before and after the change in the input (solid and dashed blue lines, respectively), for two different cases: *J* < 1/*U* and *J* > 1/*U.* As we showed above, only a facilitating population can sustain persistent activity under the first condition, while a depressing one can only do so under the second condition. If *J* < 1/*U, R* will quickly move to a new quasi–steady state as depicted in Figure 4A. Subsequent slow dynamics will push the system to the vicinity of the persistent state (solid green line in Figure 4C and black line in Figure 4E). In the *J* > 1/*U* case, there is no steady state for *R* immediately after the change in *I* (Figure 4B); thus, the activity will increase rapidly, resulting in a population spike (Figure 4D and 4F).

#### Slow dynamics.

While the initial response depends on the fast variables, the complete trajectory mainly depends on the slow *u*,*x* dynamics. Since *R* is much faster than *x* and *u*, we assume that, for each set of *x*,*u* values, it quickly reaches a steady state, determined by the first and last equations of Equation 1:

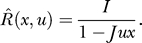
This steady state only exists when *Jux* < 1 since we are interested in suprathreshold inputs *I* > 0. The remaining equations thus reduce to the following approximate slow dynamics for *u* and *x.*

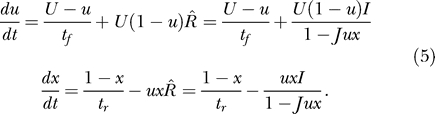



Dealing with a 2-D system instead of a 3-D one greatly simplifies the analysis. Figure 5 shows the phase space for this system in the case of strong facilitation: the *x* and *u* nullclines are depicted in blue and red, respectively, and the “forbidden” line (*Jux* = 1) is drawn in black. We consider the bistable regime (*J*_low_ < *J* < *J*_high_), such that there are three fixed points for small values of *I* (A). When the input increases, the nullclines change their configuration such that only one, high-activity fixed point remains (B), and the system begins to move toward it. If the input falls back to its baseline in a short time, the system is still in the basin of attraction of the low-activity steady state and therefore quickly returns to its original state. If the input stays on for a longer time, however, the system will cross the border between the two basins of attraction and continue its ascent to the high-activity persistent state after the removal of the stimulus. Thus, the requirement for a minimal input duration for reaching the persistent state in a facilitating population observed above (Figure 2A) is explained.

We now address the emergence of a population spike during the approach to the persistent state (Figure 2B). As explained above, the population spike occurs when the fast dynamics of *R* do not have a fixed point, which happens when the slow dynamics of *u* and *x* reach a “forbidden line” of *Jux* = 1. We therefore linearized the slow dynamics of Equation 5 near this line to determine which part of it is attracting, and which part is repulsive. The result of this analysis (see Methods) is that the attracting part is always the one that lies below the *u*-coordinate of

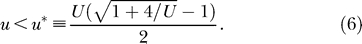



Since the condition *x* < 1 is always satisfied, this result implies that if *J* < 1/u*, the population spike cannot occur under any circumstances since the “forbidden line” is unreachable. This condition is, however, too strong, as we are interested in knowing whether the system's trajectory will reach the forbidden line during the transition to persistent activity. To find the forbidden line's basin of attraction, we numerically evaluated the separatrix between the repulsive and attractive parts by integrating the equations backward in time from the transition point *u* = u*. Figure 5C and 5D shows the repelling (solid) and attracting (dashed) parts of the forbidden line, with the basin of attraction shaded, for two different values of *J.* We found that when *J* increased above a certain numerically computed J*, the basin of attraction increased sharply and encompassed the spontaneous state, which means that the system will cross the forbidden line during its approach to the persistent state, resulting in a delayed population spike as seen in Figure 2B.

Finally, we briefly present the results of the analysis of the stability of the persistent state in the approximation of the slow dynamics. In general, stability requires that the synaptic strength exceed a certain value *J*_stab_ that can be higher than *J*_low_ (see Methods for derivation and Table 1 for formulas). If, however, the persistent state's *u*-coordinate is larger than u* (i.e., it lies across the repulsive part of the forbidden line), *J*_stab_ = *J*_low_ and the persistent state becomes stable at its inception. For zero input, *I* = 0, the persistent state can be computed analytically, and this condition is satisfied if facilitation is strong enough (see Methods for derivation):

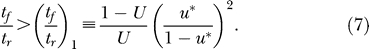



Note that if *U* is small, this inequality reduces to *t_f_* > *t_r_,* which corresponds to one of the synaptic classes found in the prefrontal cortex [12]. If

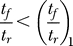
and *J*_low_ < *J* < *J*_stab_, the persistent state is unstable, and the system repeatedly reaches the forbidden line, which results in a periodic train of population spikes that we term “bursting” (see Figure 6).

### Summary of Results for a Single Subpopulation

The results obtained in the previous sections can be compactly summarized by delineating the regions in the parameter space of *J* and *t_f_*/*t_r_*, where responses of a recurrent excitatory network to a transient stimulus are qualitatively different (Figure 6). The most significant feature of the phase diagram is the emergence of three distinct regimes specified by different strengths of facilitation. A predominantly depressing population

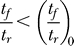
can only sustain persistent activity if *J* > *J*_high_, where it emits an instantaneous population spike after even a very short stimulus. When *t_f_*/*t_r_* is above (*t_f_*/*t_r_*)_0_, the persistent state can be reached for lower values of *J,* with or without emitting a population spike. The network exhibits an intermediate bursting activity when *J* rises above *J*_low_, and a delayed population spike for higher *J* when the persistent state becomes stable. The slow and reversible development of persistent activity allows the network to differentiate between short and long inputs. Finally, for

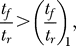
the persistent state is stable upon inception and can be reached smoothly without a population spike. We thus see that the introduction of facilitation opens new parameter regimes for persistent activity, in which the network behavior is qualitatively different from that available to a depressing population. The critical values in Figure 6 are given in Table 1.

### Network of Interacting Subpopulations

We now consider a full network comprising *P* populations (μ, ν, …), each divided into *Q* homogeneous subpopulations (α, β, …) (see Figure 1). The synapses within each subpopulation α are described by the parameters *t_f,_*_α_, *t_r,_*_α_, *U*_α_, and *J*_α_. As described in the Introduction, the connections are strongest within a subpopulation, weaker between subpopulations of the same population, and weakest between populations. The inhibitory connections are structured in a similar manner to the excitatory ones. The rate equations for the full network are derived in Methods.

**Figure 7:**
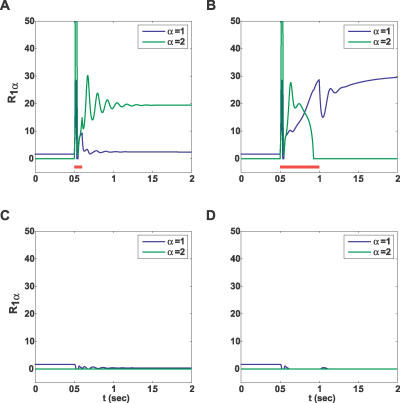
Simulation of a Full Network Two out of ten populations are shown while either a short or a long input of the same amplitude is delivered to the first population, (A) and (B), respectively. Each population consists of a facilitating and a depressing subpopulation, denoted by α = 1,2, respectively (see Methods). The resulting persistent state depends on the duration of the stimulus. (C,D) Response of a representative background population.

To illustrate the emerging properties of the full attractor neural network, we present the simulation results with *Q* = 2 subpopulations, one mainly facilitating (as in Figure 2A) and the other mainly depressing (as in Figure 2C). The inhibition to the depressing population was assumed to be stronger than that to the facilitating population, stabilizing the baseline activity of the former and allowing it to remain at rest despite suprathreshold external input. We simulated mean-field equations for a network with *P* = 10 such populations (see Methods, Equation 14). The network was presented with either a short or a long pulse to the first population. Due to cross-inhibition, only the population that receives an input exhibits a significant response (Figure 7C and 7D). Within the responding population, the behavior of the different subpopulations is qualitatively similar to that described in the previous sections (cf. Figures 2A, 2C, 7A, and 7B). The interactions between subpopulations, however, modify both the attainable steady states and the dynamics of the network. In particular, a long input drives the depressing population to its persistent state, but it only remains there until the facilitating population reaches its persistent state and inhibits the depressing one. A short input, however, can trigger the persistent state in the depressing population without allowing sufficient time for the activity in the facilitating population to build up. These two profiles of activity are reminiscent of single-neuron recordings from the lateral intraparietal area in monkeys performing working memory tasks [21]. Some neurons displayed a rapid increase in firing rate upon stimulus presentation, followed by a slow decrease during the delay period—similar to that of the depressing subpopulation's response to a long input in our simulations. Other neurons exhibited a slow ramping up of activity during the delay period, like that seen in the facilitating subpopulation. Although our simulations were not specifically tailored to reproduce the above experiments, these similarities raise the possibility that local recurrent groups of neurons with different synaptic dynamics give rise to such neural responses.

## Discussion

Recurrent excitation can maintain the activity of a cortical network even after the end of a transient stimulus. Recently, it was shown that this excitation is conveyed via a variety of dynamic synapses [12] and hence should lead to a variety of network behaviors. We showed that the introduction of strong facilitation lowers the connection strength required for the network to sustain persistent activity and enables a slow and reversible transition to persistent firing. On the other hand, networks with strong depression exhibit a rapid and transient increase in their population firing rate, termed a “population spike,” that reflects a near-coincident firing of a large number of neurons for a short duration [19,20]. Riehle et al. [22] demonstrated accurate spike synchronization in relation to events in the motor cortex of monkeys performing a delayed-pointing task. Our model predicts that multi-electrode recordings of neurons that are active in the delay period of working memory tasks can reveal transient synchronization during the transition to the persistent phase of firing. It is well-known that neurons in prefrontal cortex exhibit highly variable dynamic patterns of activity in response to sensory stimuli, spanning the range from a transient response to a gradual increase in firing rate [3,23]. Based on the analysis presented in this contribution, we suggest that this observed variability reflects the presence of embedded subpopulations of pyramidal neurons with recurrent connections of different types.

The addition of dynamic synapses to a recurrent neural network introduces two novel phenomena: population spikes and sensitivity to input duration. The first phenomenon relies on synaptic depression to terminate the increase in firing rate, while the second one requires the long timescale of synaptic facilitation. For mathematical simplicity, we chose a threshold-linear static nonlinearity for the model. We verified, however, that the qualitative behavior of the network remains the same for different static nonlinearities. Specifically, while the actual firing rates and the borders between different regimes change, neither the presence of population spikes nor the dependence of the persistent state on input duration is strongly affected by the specific nonlinearity chosen.

The analysis in this work was performed in the mean-field regime, which assumes that the contribution of fluctuations in the network is small. The cortical activity is, of course, noisy. The effects of noise can be included in mean-field models, and the resulting network behavior was shown to be qualitatively similar (see, e.g., [11]). On the other hand, there are models that attribute a more dominant role to fluctuations and at the extreme case assume a regime of activity where excitation and inhibition are balanced, thus eliminating the mean input [24,25]. It is still not clear in which regime the cortex is operating in conditions such as working memory experiments. It will be interesting to examine whether the balanced regime can be achieved with dynamic synapses and whether the qualitative results obtained in our work apply to this case.

We believe that if the different neuronal subpopulations exist in the cortical areas where persistent activity is observed, this could have far-reaching implications for the general attractor neural network theory. According to this theory, attractors are stable (persistent) states of network activity that represent long-term memory for items stored in the network (see, e.g., [5,15]). It is usually assumed that, depending on which neurons are targeted by the input, different attractors will be activated, elevating the corresponding item to a working memory state. Our results open up a possibility that even when the input targets a given fixed set of neurons, different attractors could be activated, depending on temporal features of the input, such as its duration. We illustrated this scenario by the full network simulation, where two subpopulations receive identical input (Figure 7). A short input evoked the persistent state in the depressing subpopulation, with the facilitating subpopulation remaining at a baseline activity level. A longer input of the same magnitude, however, caused a transient increase in the depressing subpopulation's firing rate followed by the transition of the facilitating subpopulation to a persistent state that inhibited the depressing one. Thus, a mutually exclusive activation of the subpopulations was demonstrated, where the input duration determines which one of them converges to a persistent state. This scenario could be extended to more complex dynamical features of the input such as its temporal frequency. We propose that this prediction could be tested experimentally in the monkey memory experiments, such as delayed saccade tasks, if the required motor response is made to depend on the temporal aspects of the cue and not only on its spatial location.

## Materials and Methods

### Derivation of rate equations.

We derive rate equations for the network of subpopulations in a standard way (see, e.g., [16]), based on the following simplifying assumptions: (1) sparse representation of stimuli resulting in nonoverlapping populations, (2) each population consists of several subpopulations with identical synaptic properties, and (3) each neuron fires a Poisson train with an instantaneous rate that is a monotonous function of its synaptic current.

We consider a network of *N* neurons (labeled *i*:1 → *N*), each receiving both synaptic and external input. The input current to a neuron, *h_i_,* changes immediately after each spike and decays exponentially to zero with a time constant *τ*. Effects of short-term plasticity are described with the model introduced in [17,18]. Each synapse (*ij*) is characterized by a set of four parameters: *J* (absolute efficacy), *U* (initial utilization parameter, analogous to release probability), and *t_f_* and *t_r_* (time course of facilitation and depression, respectively). Briefly, the running value of the utilization parameter, *u,* is facilitated every time a spike arrives, and decays to its baseline level, *U,* with the time constant *t_f_.* Correspondingly, the running fraction of neurotransmitter available, *x,* is utilized by each spike in proportion to *u* and recovers to its baseline value of 1 with the time constant *t_r_.* The neural dynamics of the network are therefore described by the following set of differential equations:

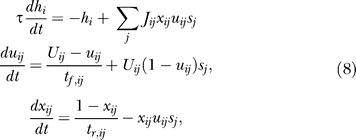
where *s_j_*(*t*) is a Poisson spike train with a rate *R_i_* that is an instantaneous monotonic function of *h_i_.*

According to the first assumption and the three-tier structuring of the network, each neuron *i* belongs to a certain population μ and subpopulation *α*:

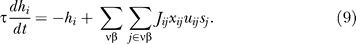



We approximate the last sum by the average of the corresponding variables over a population νβ, defining *J*_μα,νβ_, *x*_μα,νβ_, *u*_μα,νβ_, and *R*_νβ_ as corresponding average quantities:

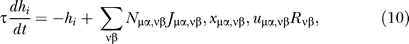
where *N*_μα,νβ_ is the average number of connections from the entire subpopulation (νβ) to a single neuron in (μα). Since the short-term synaptic dynamics in our model are uniquely determined by the presynaptic subpopulation, we can replace *x*_μα,νβ_ and *u*_μα,νβ_ by *x*_νβ_ and *u*_νβ_, respectively. We absorb the factor *N*_μα,νβ_ into the definition of *J*_μα,νβ_ and thus have:

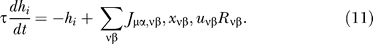



Note that *h_i_* only depends on μα and νβ, we can thus write

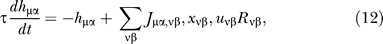
where *R*_νβ_ is taken to be a threshold-linear function of *h*_νβ_ for simplicity, yielding the following set of mean-field equations:

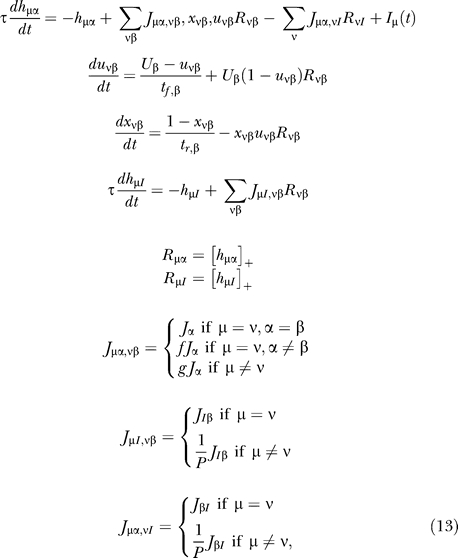
where [*x*]_+_ ≡ max(*x*, 0), *f* and *g* are numerical factors that define the relative scaling of synaptic strength in the three-tier structure described above, and (ν*I*) is the inhibitory subpopulation associated with population ν.

### Steady states of the homogeneous subpopulation.

By demanding a steady state in Equation 1, we get the following equation for *R*:

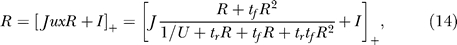
which for *I* ≤ 0 gives *R* = 0 and up to two more solutions, and for positive *I* up to three positive solutions. The value *J*_low_, for which a positive solution appears at *I* = 0, can be found by looking at the maximal value of *Jux* in the steady state, and by demanding that it be at least 1:

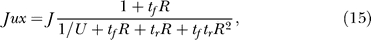


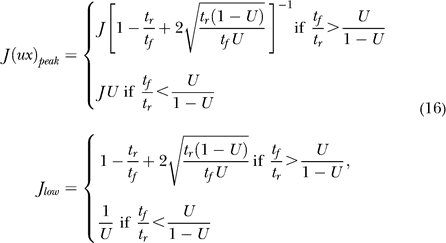
 where the condition *t_f_/* *t_r_* > *U*/(1 − *U*) is simply the condition for *Jux* to have a maximum for positive *R*. Note that the same condition ensures that, for small *R, Jux* is an increasing function of *R,* and thus *JuxR* is convex leading to Equation 3.

### Steady states of the 2-D approximation.

As mentioned in the text, we use the separation of timescales to define 2-D dynamics on the slow variables *x* and *u.* Equation 5 allows us to derive formulas for the nullclines:

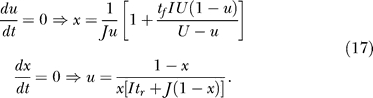



### Stability of the persistent state.

This approximation also allows us to test the stability of the persistent state for small positive inputs. If *I* = ɛ is the input, then first order we have for *x:*

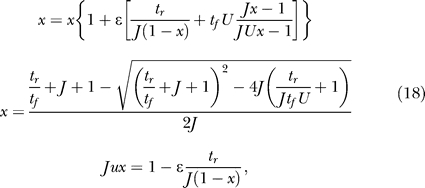
and for *u*:

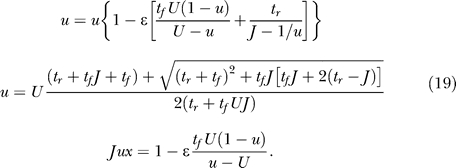



This solution is the one corresponding to the persistent state. There is one unstable solution (obtained by taking the plus solution of the *x* quadratic equation) and the spontaneous state solution

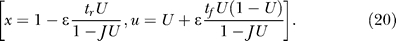



We now consider the linearized dynamics:

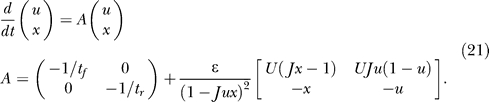



Since 1 − *Jux* ≈ ɛ, the second matrix dominates as ɛ → 0, and we have:

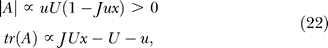
with the condition for stability being *tr*(*A*) < 0:

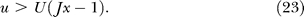



For ɛ → 0, using *Jux* ≈ 1, this condition reduces to

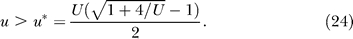



For *J* = *J*_low_, there is only one solution to the steady state, and hence from Equation 19,

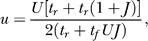
and by combining with Equation 16, we get

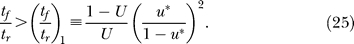



If *t_f_*/*t_r_* is smaller than (*t_f_*/*t_r_*)_1_, the persistent state is not stable for *J* = *J*_low_, and solving Equation 24 leads to the stability condition:

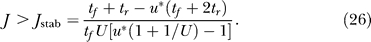



### Stability of the forbidden line.

The 2-D approximation is ill-defined when *Jux* = 1, which calls for an analysis of the conditions for this line being attractive. Consider a point *s̄*, *ū* on this line, and a point *x, u* near it:

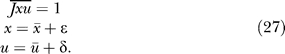



To first order in ɛ,δ we have:

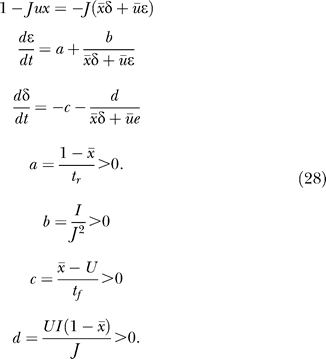



We are interested in the dynamics for *w* = *s̄*δ + *ū*ɛ, which is the distance from the forbidden line (with negative sign):

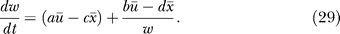



For small *w,* the condition for the line being attractive is

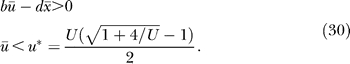
Note that this is the same u* from Equation 24. This condition also defines a minimal *J* = 1/u* for a population spike to be attainable. This value is only a lower bound on the actual J* for which population spikes appear, since these depend on the trajectory actually entering the forbidden line's basin of attraction. We were able to calculate J* numerically by integrating backward in time from (1/Ju*, u*) and increasing *J* until the separatrix crossed the spontaneous state.

### Simulation parameters.

Table 2 shows the four parameter sets that were used throughout the figures. Any deviation from these values is described in the text or captions. For the network simulation in Figure 7, we used Equation 13 with the following parameters: *f* = 0.1, *g* = 0.01, *J_I_*_α_ = (0.5,0.4), and *J*_α*I*_ = (0.3,0.7). The two subpopulations used parameter sets A and C (with *J* = 4.5).

## Abbreviations

attractor: persistent activity states in neural networks and dynamic synapses with short-term synaptic plasticity
