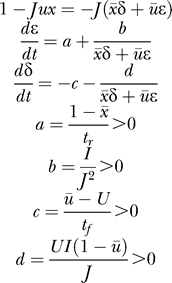# Correction: Persistent Activity in Neural Networks with Dynamic Synapses

**DOI:** 10.1371/journal.pcbi.0030104

**Published:** 2007-05-25

**Authors:** Omri Barak, Misha Tsodyks

In *PLoS Computational Biology,* volume 3, issue 2: doi: 10.1371/journal.pcbi.0030035

Equation 28 had incorrect symbols in lines 3, 6, and 7. Here is the correct Equation 28: